# HPV vaccine for adolescents in China: what is the next step?

**DOI:** 10.1016/j.lanwpc.2026.101810

**Published:** 2026-01-30

**Authors:** The Lancet Regional Health – Western Pacific

Beginning on Nov 10, 2025, two free doses of bivalent human papillomavirus (HPV) vaccination are being rolled out across China for girls who have reached 13 years of age. China has the world's highest numbers of cervical cancer cases and the second largest number of cervical cancer deaths. But coverage of the first dose of HPV vaccines, one of the most cost-effective measures to reduce cervical cancer incidence, in girls aged 9–14 years was only 4% in 2022, significantly behind the global average of 61.6%. The introduction of HPV vaccines in its National Immunisation Programmes represents a major landmark and a strong political will of China to eliminate cervical cancer. Despite the successful experience of delivering free HPV vaccines in a few pilot cities, there are uncertainties and challenges to implement HPV vaccination at a national level.

HPV vaccines were first introduced in China on the private market in 2016. Like many other countries, confidence in vaccination, accessibility and affordability of vaccines, and social norms have hindered HPV vaccine uptake rate in China. The dynamic and complex nature of HPV vaccine hesitancy in China has been influenced by modifiable factors such as lack of awareness or safety and efficacy concerns on domestic vaccines; more resistant reasons related to cultural perceptions and low trust. The current gender-specific focus on the HPV vaccine could further enhance social stigmatisation and discrimination toward girls who receive it. From a supplier perspective, readiness of the health-care system to effectively deliver the new vaccine at scale remains to be tested. China has not introduced new vaccines in its National Immunisation Programmes since 2008. There are knowledge gaps on HPV vaccines among health-care workers, and vaccine delivery at the grass levels could pose challenges. Past experiences in other national immunisation programmes indicate that issues such as substantial geographical variations in quality and quantity of vaccination procedures and distribution are likely to recur. Additionally, financial sustainability of the maintenance and potential expansion of the immunisation programme remains uncertain.

China can gain insights and practical guidance from its previous pilot programmes and experiences from other countries, but it's crucial to establish context-specific solutions to address the unique challenges of national implementation. To improve public confidence in HPV vaccines and the national programme, gender-neutral knowledge of HPV infections and the benefits of HPV vaccination need to be disseminated. Details on administration and side effects should be transparently communicated to the public at this very early implementation stage. Particularly, safety and effectiveness data of domestic vaccines and how they were assessed should be explained. Given that a domestic 9-valent vaccine has been approved in 2025, which demonstrated non-inferior effectiveness compared with the imported 9-valent vaccine and at a much lower price, it may be worthwhile to consider and assess the feasibility and cost-effectiveness of a nationwide free 9-valent based immunisation programme.

To ensure sustainability and equality of the national HPV immunisation campaign, a convenient and coordinated vaccination mechanism and a comprehensive immunisation information system is needed. International experiences have shown that standardised school-based immunisation programmes are essential for the success of the national programmes. In China, school-based immunisation programmes could further take local administrative capacity into consideration to overcome regional disparities. These can be complemented by coordination with community and primary care facilities to ensure vulnerable groups such as out-of-school adolescents being adequately reached. In these programmes, the responsibilities of all participating sectors should be clearly defined, and the quality of vaccination services should be ensured. Moving forward, there are further steps to popularise the vaccine and maximise the benefits of HPV vaccination: incorporating boys, refining the age groups, and optimising the immunisation strategy. Gender-neutral vaccination programmes could be implemented to close the gender gap. Tens of millions of women who already missed the optimal window for prevention should be recommended and financially supported to receive catch-up HPV vaccinations despite the diminished protection. Tailored HPV immunisation recommendations such as Australia's immunisation schedule based on different ages and risk groups could be established to optimise the effectiveness of vaccination.

During this year's Cervical Cancer Prevention Week, we celebrate the good news from China to vaccinate their adolescents against HPV. However, this is only the first step. Continued political determination, multisectoral collaborations, innovative strategies, and sustained funding and resources are needed to eliminate cervical cancer for all.

